# Systemic Disease and Ocular Comorbidity Analysis of Geographically Isolated Federally Recognized American Indian Tribes of the Intermountain West

**DOI:** 10.3390/jcm9113590

**Published:** 2020-11-07

**Authors:** Patrice M. Hicks, Benjamin Haaland, Michael Feehan, Alan S. Crandall, Jeff H. Pettey, Elizabeth Nuttall, William Self, Mary Elizabeth Hartnett, Paul Bernstein, Albert Vitale, Akbar Shakoor, Julia P. Shulman, Sandra F. Sieminski, Ivana Kim, Leah A. Owen, Maureen A. Murtaugh, Albert Noyes, Margaret M. DeAngelis

**Affiliations:** 1 Department of Ophthalmology and Visual Sciences, University of Utah, Salt Lake City, UT 84132, USA; patrice.hicks@hsc.utah.edu (P.M.H.); alan.crandall@hsc.utah.edu (A.S.C.); jeff.pettey@hsc.utah.edu (J.H.P.); elizabeth.nuttall@hsc.utah.edu (E.N.); wjself@moffatfarm.com (W.S.); me.hartnett@hsc.utah.edu (M.E.H.); paul.bernstein@hsc.utah.edu (P.B.); albert.vitale@hsc.utah.edu (A.V.); akbar.shakoor@hsc.utah.edu (A.S.); Leah.Owen@hsc.utah.edu (L.A.O.); albert.noyes@mosaicmedical.org (A.N.); 2 Department of Population Health Sciences, University of Utah, Salt Lake City, UT 84108, USA; Ben.Haaland@hsc.utah.edu (B.H.); maureen.murtaugh@hsc.utah.edu (M.A.M.); 3 Kantar, LLC, New York, NY 10007, USA; michael.Feehan@kantar.com; 4 Department of Ophthalmology, New York Medical College, Valhalla, NY 10595, USA; jshulman03@gmail.com; 5 Department of Ophthalmology, Jacobs School of Medicine and Biomedical Engineering, University at Buffalo SUNY, and the VA Western New York Healthcare System, Buffalo, NY 14215, USA; sandrafe@buffalo.edu; 6 Retina Service, Harvard Medical School, Massachusetts Eye and Ear, Boston, MA 02114, USA; ivana_kim@meei.harvard.edu

**Keywords:** epidemiology, hypertension, diabetes, retinopathy, American Indian, isolated populations

## Abstract

Background: The American Indian Navajo and Goshute peoples are underserved patient populations residing in the Four Corners area of the United States and Ibupah, Utah, respectively. Methods: We conducted a cross-sectional study of epidemiological factors and lipid biomarkers that may be associated with type II diabetes, hypertension and retinal manifestations in tribal and non-tribal members in the study areas (n = 146 participants). We performed multivariate analyses to determine which, if any, risk factors were unique at the tribal level. Fundus photos and epidemiological data through standardized questionnaires were collected. Blood samples were collected to analyze lipid biomarkers. Univariate analyses were conducted and statistically significant factors at *p* < 0.10 were entered into a multivariate regression. Results: Of 51 participants for whom phenotyping was available, from the Four Corners region, 31 had type II diabetes (DM), 26 had hypertension and 6 had diabetic retinopathy (DR). Of the 64 participants from Ibupah with phenotyping available, 20 had diabetes, 19 had hypertension and 6 had DR. Navajo participants were less likely to have any type of retinopathy as compared to Goshute participants (odds ratio (OR) = 0.059; 95% confidence interval (CI) = 0.016–0.223; *p* < 0.001). Associations were found between diabetes and hypertension in both populations. Older age was associated with hypertension in the Four Corners, and the Navajo that reside there on the reservation, but not within the Goshute and Ibupah populations. Combining both the Ibupah, Utah and Four Corners study populations, being American Indian (*p* = 0.022), residing in the Four Corners (*p* = 0.027) and having hypertension (*p* < 0.001) increased the risk of DM. DM (*p* < 0.001) and age (*p* = 0.002) were significantly associated with hypertension in both populations examined. When retinopathy was evaluated for both populations combined, hypertension (*p* = 0.037) and living in Ibupah (*p* < 0.001) were associated with greater risk of retinopathy. When combining both American Indian populations from the Four Corners and Ibupah, those with hypertension were more likely to have DM (*p* < 0.001). No lipid biomarkers were found to be significantly associated with any disease state. Conclusions: We found different comorbid factors with retinal disease outcome between the two tribes that reside within the Intermountain West. This is indicated by the association of tribe and with the type of retinopathy outcome when we combined the populations of American Indians. Overall, the Navajo peoples and the Four Corners had a higher prevalence of chronic disease that included diabetes and hypertension than the Goshutes and Ibupah. To the best of our knowledge, this is the first study to conduct an analysis for disease outcomes exclusively including the Navajo and Goshute tribe of the Intermountain West.

## 1. Introduction

Underserved and rural populations have unique paradigms for both disease risk and disease prevention. Part of this can be attributed to the challenges to accessing quality healthcare and preventative care services because of their geographic location. Accordingly, these populations may offer more thorough insight into understanding the risks of certain diseases as well as the etiology in a more homogenous setting not found in other settings [1,2,3]. This is due to the fact that there is less likely genetic and environmental heterogeneity which may increase the ability to identify common mediators of disease. Currently, there are 573 tribes that are considered federally recognized within the United States and receive funding and services from the Bureau of Indian Affairs (BIA, Washington, DC, USA) [4,5,6]. In the United States, American Indian reservations are often in geographically rural areas [4,7,8]. Residents who reside on a reservation can experience significant difficulties in accessing both healthcare and preventative services [4,7,8]. Challenges in receiving adequate healthcare and preventative services also include obtaining routine eye examinations, which could alert to the presence of systemic diseases such as diabetes and hypertension [9,10,11,12,13,14]. Moreover, for those that live in geographically isolated areas, it is known that there are challenges not only to accessing adequate healthcare and preventative care services but also resources to maintain a healthy lifestyle [15,16]. Resources include nutritious foods to help prevent and manage diseases such as diabetes, hypertension and dyslipidemia, which can increase the likelihood of morbidity and mortality [17]. Those who reside on reservations may experience food insecurity because of a lack of access to high-quality nutritious food. Both food and nutritional security are fundamental human needs as well as crucial social determinants of health [18,19,20]. While it is broadly accepted that experiencing food insecurity can also increase the risk for obesity and diseases such as hypertension and diabetes, this correlation has been specifically observed among the American Indian population in general [17,21,22].

There is not much information related to the different clinical, environmental and demographic risk factors for ocular disease within the American Indian population at the tribal level. Specifically, there is a lack of information showing the prevalence of ocular disease for individual tribes within this population, as well as secondary ocular diseases that occur due to systemic disease including hypertensive retinopathy and diabetic retinopathy. Available research findings pertaining to disease manifestation in the retina could lead to diagnosis of both diabetic and hypertensive retinopathy as these diseases co-segregate with the systemic diagnosis of diabetes and hypertension. The prevalence of diabetes and hypertension is well known to be higher within the American Indian population as compared to non-Hispanic whites [23,24]. Within the American Indian population, there is drastically more mortality and morbidity due to diabetes and hypertension as compared to non-Hispanic whites [4,25,26,27]. Within the American Indian population in the United States, research has shown that the prevalence of diabetes ranges from 12% to 70% in those who are aged 20 to 74 years of age [28,29,30,31]. Prevalence also varies by American Indian tribe [28,29,30,31]. Another study found that the prevalence of hypertension in the American Indian population in the United States ranges from 27% to 44% and, similarly to diabetes, varies by American Indian tribe, with an average age of 40.8 in the study population [32]. Due to the higher prevalence of these chronic diseases, researchers may see that the Native American population has higher prevalence of hypertensive and diabetic retinopathy.

Within the working population, as they age, the majority of blinding eye diseases that affect the back of the eye (retina) have been linked to retinopathy. This can manifest as either hypertensive or diabetic retinopathy. These two retinopathies have been linked to environmental risk factors including dyslipidemia, poor nutrition, hypertension and diabetes [33,34,35]. In some instances, these diseases can manifest within the eye before they manifest systemically [36,37,38,39,40,41,42]. These eye diseases can be diagnosed by a color fundus photo, which is a picture of the retina [36,37,38,39,40,41,42]. Patients may not know that they have hypertension and/or diabetes until they receive a retinal examination by an eye care professional [43,44,45]. In addition, previous studies have also observed in both American Indian and Caucasian populations a link between both the severity of retinopathy and risk for cardiovascular outcomes such as stroke and heart disease [46,47,48,49].

Previous studies that have examined eye disease prevalence within federally recognized American Indian tribes include the Family Investigation of Nephropathy and Diabetes (FIND-eye) study [49], the Strong Heart Study (SHS) [46,47] and the Population Architecture Using Genomics and Epidemiology (PAGE) study [48]. The SHS also reported on the prevalence of hypertension within their study population, which was stated to be 35.7% [47]. These studies have been informative for public health because they have provided useful information with respect to visual impairment within the American Indian tribes of the United States at an aggregate level, but at the individual tribal level. An informative way to achieve these needs is by designation of each individual tribe that each study participant may belong to [50]. Former studies aiming to determine the prevalence of blinding eye disease in the American Indian population generalized that diabetic retinopathy was found within their study population, suggesting that hypertensive retinopathy was either not found or not as prevalent within their study population. These studies did not outline which specific tribe(s) participated in their studies [46,47,48,49]. Previous research has reported that diabetic retinopathy in the American Indian population ranges from 17.7% to 49% for non-proliferative diabetes in multiple population-based epidemiological studies [51]. Of the 573 federally recognized tribes in the United States, 24 comprise the Intermountain West, where the Confederate tribes of the Goshute reservation and the Navajo Nation reside. The U.S. Department of Agriculture defines the Intermountain West as a western region of the United States that includes the Columbia River Basin and Snake River Plateau in the northwest, the Great Basin in Nevada and Western Utah and the Colorado Plateau in the Four Corners area of Utah, Arizona, New Mexico and Colorado [52].

The Confederate tribes of the Goshute reservation, for whom we have previously published risk factors for retinopathy, reside within the Intermountain West of the United States and span the states of Nevada and Utah. The Confederate tribes of the Goshute reservation are considered one of the most geographically isolated of all the federally recognized tribes in the United States [53,54]. The Navajo Nation, also of the Intermountain West, is located in the Four Corners region of the United States, spanning 27,000 square miles [55]. The region is the intersection of the states Utah, Arizona, Colorado and New Mexico. The Navajo Nation is considered the most populous American Indian tribe in the United States, with a population of 156,823. A previous cross-sectional study included a population of 60 Navajo American Indians living in Northeastern Arizona. The study participants had non-insulin-dependent diabetes which was confirmed by both hospital records and fasting plasma glucose concentration. This study reported a 29% prevalence of diabetic retinopathy, a diagnosis that was made by ophthalmic examination [56]. Unfortunately, much of the research about ophthalmic disease in the Navajo population of the Intermountain West is from the 1990s [57].

Our study, the Supporting Prediction and Prevention Blindness Project (SPBPP), aimed to identify the clinical, demographic and environmental risk factors for blinding eye disease and systemic disease in the Navajo and Goshute populations. We studied these populations individually as broad level research may not create the appropriate changes needed to address individual tribal needs [58,59]. By studying the Navajo located at the Four Corners, 557 miles southeast of Ibupah, Utah, where the Goshute American Indians reside, we sought to determine what, if any, similarities or differences existed, given that these individual tribes are both part of the Intermountain West. We also sought to determine the prevalence of both systemic and retinal disease within both study populations, with the goal being to provide information to enhance disease interventions and treatments for these specific American Indian tribes. Due to these two tribes’ isolations and the remoteness of their rural areas, this approach holds the potential to further explore how the environment impacts a person’s overall health as well as the cultural and social factors that may impact healthy aging and wellbeing. This was further supported by medical records. We believe this study has implications for understanding disease risk as it pertains to hypertension and diabetes, as well as preventing these diseases and their ocular comorbidities. Studying the epidemiology within these populations may also provide broader implications for understanding complex chronic diseases not only in other American Indian populations but in other geographically isolated populations. This is the first study to examine differences for systemic and eye disease between the two individual tribes, the Navajo Nation and the Confederate tribes of the Goshute reservation.

## 2. Experimental Section

### 2.1. Study Cohort

The study protocol was reviewed and approved by the Institutional Review Board at the University of Utah and the Navajo Nation Institutional Review Board. This study conformed to the tenant of the Declaration of Helsinki. Study participants were enrolled in this study after giving written informed consent. The study team visited the Utah Navajo Health System (UNHS) and met with the Board of Directors, who represent each of their individual community chapters within the Navajo American Indian population. There are 156,823 Navajo tribal members living on the reservation in the Four Corners region [60]. The study team explained the importance of the study and the benefits of the study to the Board of Directors. The Board of Directors of the Utah Navajo Health System provided valuable counsel pertaining to the study. In addition, they expressed interest in continued assistance and involvement as liaisons between the study project and their respective Navajo American Indian communities. Study participants were recruited within the Utah Navajo Health System located in the in the Four Corners region of the Intermountain West. We recruited from all members of the Navajo Nation community that expressed an interest in the study. Therefore, this is a convenience sample study design. In total, 51 study participants were considered for analysis of the 82 participants consented to participate due to completeness of obtained data. The Ibupah, Utah study population consisted of participants from the Confederate tribes of the Goshute reservation and Ranchers of Northern European descent that resided near Ibupah, Utah. The tribal membership for the Confederate tribes of the Goshute reservation is 409 [61]. We had similar cohort recruitment for the Ibupah, Utah study cohort. The methods for establishing this study population have been previously published [51]. Our analysis specifically examined factors of both morbidity and mortality in both tribes. Blood pressure and body mass index were obtained from each of the study participants the day of their visit. Diabetes status and cholesterol were obtained from study participants’ medical records. In addition, blood samples were collected to analyze lipid biomarkers. Furthermore, intraocular pressures and colored digital retina fundus photographs were obtained for each of the study participants. Intraocular pressure was measured using the Goldmann applanation tonometry. Finally, all of the study participants completed a validated standard epidemiological questionnaire administered by the study team. We employed a detailed standardized epidemiological questionnaire that assessed clinical, demographic and environmental risk factors of morbidity and mortality along with assessment of cardiovascular disease biomarkers.

### 2.2. Phenotypic Analysis

We have previously published details on how the Goshute American Indian population was evaluated for blinding eye disease using a standardized methodology [50]. This same methodology was used to evaluate the Navajo American Indian population within this study for comparison to the Goshute American Indian population, including a comprehensive eye examination performed by an ophthalmologist. All study participants had non-mydriatic fundus photos obtained from both of their eyes. The digital retina scanner that was used was a Canon CR-2 digital retina camera with EOS camera technology, manufactured by Canon Inc., Irvine, California, United States of America. The photos were then evaluated by at least seven independent ophthalmologists with retinal specialization. Each of the photographs was evaluated for the presence of retinal disease. The presence, absence and severity of retinal disease were evaluated by at least seven retina specialists using standardized methods to evaluate retinal disease. If there was an inconsistent diagnosis of a retinal disease for a participant, the inconsistency was resolved through either direct and/or phone communication between at least two of the evaluating ophthalmologists. Blood samples were used to determine abnormal amounts of cholesterol, triglycerides and fat phospholipids in the participant’s blood to determine a diagnosis of dyslipidemia.

### 2.3. Statistical Analysis

Factors associated with comorbidities of retinal diseases were evaluated to determine the degree of any statistical associations. Univariate (unadjusted model) and multivariate (adjusted model) analyses were conducted for both outcomes of a diagnosis of diabetes and/or hypertension. An analysis of diabetes was conducted in the total study population, followed by a diabetic diagnosis outcome analysis of only the Navajo American Indian population. We conducted another analysis of the high blood pressure outcome in the total study population, followed by the same analysis restricted to the Navajo American Indian population. Univariate and multivariate analyses were also conducted for the outcome of the diabetic retinopathy in the total study population. Univariate logistic regression analysis was conducted and further justified using Fisher’s exact test given the relatively limited sample size within this underserved isolated population [62,63,64]. The variables age, body mass index, sex, American Indian descent, tribe location, diabetes and hypertension were entered into the model for the retinopathy meta-analysis that included both the geographically isolated populations of the Four Corners of the Intermountain West and Ibupah, Utah. The same variables were used for the outcome of hypertension and diabetes, excluding the outcome variable of interest. The variables age, body mass index, sex, tribe location, diabetes and hypertension were entered into the model for the retinopathy meta-analysis that included both the Navajo American Indian and Goshute American Indian study populations. The same variables were used for the outcome of hypertension and diabetes, excluding the outcome variable of interest. To determine the most predictive model for our observed phenotypes for both blinding eye disease and chronic systemic conditions in the individual populations, i.e., Four Corners of the Intermountain West, Navajo American Indian tribe, Ibupah, and the Goshute American Indian populations, a univariate analysis was conducted and only risk factors with an association at *p* < 0.10 were included in the multivariate model (DeAngelis 2004). [65] Risk factors from the multivariate analysis were only considered statistically significantly associated with the chronic disease outcome or eye disease outcome at *p* < 0.05.

## 3. Results

### 3.1. Demographic Factors

In total, 51 individuals from the Four Corners region for whom we had compete study data were included in this research study (Table 1). Demographic factors for this population are presented in Table 1. The study population was predominantly older than 45 years of age, female and had a high level of hypertension, diabetes, obesity and dyslipidemia.

There were 31 diabetics in our study population (Table 2). The demographic factors for diabetics are presented in Table 2. Diabetic participants were evenly distributed between men and women. Diabetic participants tended to be older, have hypertension and be American Indian.

There were 26 participants with hypertension in our study population (Table 3). Hypertensive participants were almost evenly distributed between men and women. Hypertensive participants tended to be older, diabetic and American Indian.

### 3.2. Factors Associated with Diabetes in the Four Corner and Navajo American Indian Populations

A univariate analysis was conducted for the outcome of diabetes within the total study population of the Four Corners of the Intermountain West (Table 4). We found that hypertension ((odds ratio) OR = 9.8, 95% (confidence interval (CI) = 2.6–37.4), age greater than or equal to 45 years old (OR = 3.6, 95% CI = 1.8–22.1) and female sex (OR = 3.2, 95% CI = 0.9–11.0) were statistically significantly associated with an outcome on diabetes at *p* < 0.10. In the multivariate model, both female sex (adjusted odds ratio)AOR = 3.3; 95% CI = 0.8–14.0) and age greater than or equal to 45 years (AOR = 2.5; 95% CI = 0.5–12.1) were not significantly associated with diabetes at *p* < 0.05. Hypertension remained statically significant (AOR = 6.2; 95% CI = 1.3–30.8).

A univariate analysis of diabetic factors in the Navajo American Indian study population of the Four Corners of the Intermountain West found that the factors hypertension (OR = 22.5, 95% CI = 4.1–123.8) and age greater than or equal to 45 (OR = 8.8, 95% CI = 2.2–35.0) were significantly associated with diabetes at *p* < 0.10. In the multivariate analysis, age greater than or equal to 45 was no longer significant for diabetes (AOR = 3.1; 95% CI = 0.6–16.). Hypertension remained significantly associated with diabetes (AOR = 13.6; 95% CI = 2.2–84.3). (Table 5)

### 3.3. Hypertension Factors in the Four Corners and Navajo American Indian Study Populations

We found that diabetes (OR = 9.8, 95% CI = 2.6–37.4), age greater than or equal to 45 years (OR = 16.3, 95% CI = 3.8–70.7) and dyslipidemia (OR = 3.4, 95% CI = 1.0–11.9) were significantly associated with hypertension in the total study population of the Intermountain West at *p* < 0.10. (Table 6) In the multivariate model, both age greater than or equal to 45 years (OR = 11.5, 95% CI = 2.3–58.5) and diabetes (OR = 5.9, 95% CI = 1.2–28.1) remained statically associated with hypertension in the total study population of the Four Corners of the Intermountain West. Dyslipidemia was no longer significantly associated with hypertension in the total study population of the Four Corners of the Intermountain West (OR = 3.6; 95% CI = 0.7–19.1).

The univariate analysis found that age greater than or equal to 45 years of age (OR = 14.3, 95% CI = 3.2–64.6), dyslipidemia (OR = 3.1, 95% CI = 0.9–11.3) and diabetes (OR = 22.5, 95% CI = 4.1–123.8) were statistically significantly associated with a diagnosis of hypertension in the Navajo American Indian study population of the Intermountain West (Table 7). After conducting the multivariate analysis, we found that both a diagnosis of diabetes (OR = 12.4, 95% CI = 1.9–79.0) and age greater than or equal to 45 years old (OR = 8.1, 95% CI = 1.4–46.8) were both still significant factors associated with the outcome of hypertension in the study population of the Navajo American Indian population of the Four Corners of the Intermountain West, while dyslipidemia was not (OR = 2.8, 95% CI = 0.5–16.6).

### 3.4. Diabetic Retinopathy Factors in the Four Corners and Navajo American Indian Study Populations

The ophthalmologists within our study used the participants’ dilated fundus photographs to determine which eye disease or diseases were prevalent within our study population of both the total study population and the Navajo American Indian study population of the Four Corners of the Intermountain West. The prevalent eye diseases within our population are presented in Table 8.

All participants in the study population from the Four Corners of the Intermountain West with diabetic retinopathy were age 45 years old or greater, had hypertension and also had diabetes. In the univariate analysis, we found that body mass index greater than or equal to 30 was statistically significantly associated with a diagnosis of hypertension in the Navajo American Indian population (OR = 0.2, 95% CI = 0.0–1.1). All of the Navajo American Indian study participants with diabetic retinopathy. Body mass index greater than or equal to 30 was no longer statically significant in the multivariate model (OR = 0.2, 95% CI = 0.0–1.4) (Table 9).

### 3.5. Demographic Factors in the Ibupah, UT and Goshute American Indian Study Populations

We also observed risk factors for diabetes and hypertension in another underserved, federally recognized tribe of the Intermountain West, the Confederate tribes of the Goshute reservation. We had 50 Goshute American Indians participate, constituting part of the total study population of Ibupah, Utah (Table 10). Demographic factors of this study population are presented in Table 10.

### 3.6. Diabetes Factors in the Ibupah, UT and Goshute American Indian Study Populations

A univariate analysis of factors associated with diabetes in the total study population in Ibupah, Utah found that hypertension (OR = 5.0, 95% CI = 1.6–16.3) and being American Indian (OR = 5.3, 95% CI = 0.9–61.2) were statistically significantly associated with diabetes at *p* < 0.10. In the multivariate analysis, hypertension (OR = 5.3, 95% CI = 1.6–16.3) remained statistically significant, while being American Indian (OR = 7.5, 95% CI = 0.8–68.4) did not (Table 11).

A univariate analysis was conducted to determine factors that were statically significant with a diagnosis of diabetes in the study population of the Goshute American Indian population of Ibupah at P < 0.10 found that the factors hypertension (OR = 6.9, 95% CI = 1.8–25.8) and age greater than or equal to 45 years (OR = 3.0, 95% CI = 0.9–10.0) were statistically significantly associated with diabetes. The multivariate analysis found that age greater than or equal to 45 years was no longer statistically significant (OR = 2.2; 95% CI = 0.6–8.2). Hypertension remained statistically significant in association with diabetes in the study population of the Goshute American Indian population (OR = 6.1; 95% CI = 1.6–23.4) (Table 12).

### 3.7. Hypertension Factors in the Ibupah, UT and Goshute American Indian Study Populations

In the univariate analysis, we observed that diabetes (OR = 5.0, 95% CI = 1.6–16.3), age greater than or equal to 45 years (OR = 2.8, 95% CI = 0.9–9.2) and a body mass index of 30 or greater (OR = 3.4, 95% CI = 0.8–13.8) were statistically significant at *p* < 0.10 (Table 13). In the multivariate model, diagnosis of diabetes (OR = 3.9, 95% CI = 1.1–13.6) remained statistically associated with a diagnosis of hypertension in the total study population of Ibupah, Utah in the multivariate logistic regression. Body mass index of greater than or equal to 30 (OR = 2.7; 95% CI = 0.6–11.8) and age greater than or equal to 45 years (OR = 2.4; 95% CI = 0.6–8.9) were no longer statistically significantly associated with hypertension in the multivariate analysis.

All participants with a diagnosis of hypertension in the study population of Goshute American Indians in Ibupah, Utah had a body mass index of greater than or equal to 30. In addition, none of the study population of Goshute American Indians in Ibupah, Utah had a body mass index of greater than or equal to 25 and less than 30. Diabetes was associated with a diagnosis of hypertension (OR = 6.9, 95% CI = 1.8–25.8). Diabetes (OR = 5.6, 95% CI = 1.3–26.3) remained statistically significant in the multivariate model (Table 14).

### 3.8. Combined Analysis of Diabetes in the Ibupah, Utah and Four Corners Populations

In the adjusted multivariate model, hypertension (OR = 6.1, 95% CI = 2.2–16.8), location (OR = 3.0, 95% CI = 1.1–7.9) and being American Indian (OR = 7.2, 95% CI = 1.3–39.4) were statistically associated with diabetes in the combined study population. Those living in the Four Corners region were more likely to have diabetes (Table 15).

In the adjusted multivariate model, hypertension was statistically associated with diabetes in the overall combined American Indian study population (OR = 7.9, 95% CI = 2.7–23.0) (Table 16).

### 3.9. Combined Analysis of Hypertension in the Ibupah, Utah and Four Corners Populations

Within this multivariate analysis, we found that diabetes (OR = 6.1, 95% CI = 2.2–16.9) and age greater than or equal to 45 years of age (OR = 5.1, 95% CI = 1.8–14.3) was statically significantly associated with hypertension in the overall combined population (Table 17).

A similar combined population analysis was conducted in solely the American Indian study population, which found that both diabetes (OR = 7.8; 95% CI = 2.7–22.8) and age greater than or equal to 45 years old (OR = 3.8; 95% CI = 1.3–11.4) were statistically associated with a diagnosis of hypertension (Table 18).

### 3.10. Combined Analysis of Retinopathy in the Ibupah, Utah and Four Corners Populations

In the multivariate analysis, we found, after adjusting for all risk factors, that both location (OR = 0.1, 95% CI =0.0–0.2) and hypertension (OR = 3.7, 95% CI = 1.1–12.4) were statistically associated with retinopathy in the overall study population at *p* < 0.05. Those residing in the Four Corners were less likely to have retinopathy as compared to those in Ibupah, Utah (Table 19).

In our analysis, for the outcome of retinopathy in the overall American Indian study population from both study populations, we found that location was statistically significantly associated with a diagnosis of retinopathy in the overall American Indian study population. The Navajo American Indian study participants had decreased odds of having a diagnosis of retinopathy as compared to the Goshute American Indian study participants (OR = 0.059; 95% CI = 0.016–0.223) (Table 20).

## 4. Discussion

The Colorado Plateau, where four states come together in the United States, is where the largest Navajo Nation resides. The western part of Utah, where Ibupah is located, is where the Confederate tribes of the Goshute Reservation reside. Therefore, both of the populations studied reside in the United States’ Intermountain West, albeit in different locations, approximately 557 miles apart. Native American Indians living in Ibupah, Utah, were more likely to have any retinopathy than those living in the Four Corners of the Intermountain West. Additionally, Goshute American Indians were more likely to have retinopathy as compared to Navajo American Indians. However, it should be noted that 62.2% of the Navajo study population had diabetes mellitus and that the prevalence of diabetic retinopathy was 13.8%. This could be due to better access to a healthcare system in the Four Corners of the United States.

Diabetes mellitus does not always progress into diabetic retinopathy because of greater access to care, particularly compared to other Native American tribes. We have previously demonstrated that while the Goshute Native American Tribe in Ibupah had a prevalence of diabetes mellitus at 38%, there was only 10% with diabetic retinopathy while concurrently having far less access to healthcare (at least two hours’ drive) compared to the Navajo Native Americans. In terms of diabetes, those with hypertension were more likely to have the disease in both of the study populations [50]. However, the prevalence was more significant for the Navajo. Within the Navajo and Four Corners populations, older age was associated with diabetes. When observing hypertension, those with diabetes were more likely to have the disease, within both populations, with a greater prevalence in the Navajo population. The Goshute and Navajo population had a similar BMI of greater than or equal to 30, with 72% and 68.9%. Obesity was considered a risk factor for diabetic retinopathy in our study as previous literature supports this. A study that included 156 participants demonstrated that higher body weight was the most significant risk factor for diabetic retinopathy than age, diabetes management or duration of diabetes [66].

Moreover, another study of 492 study participants showed that higher BMI was statistically associated with diabetic retinopathy [67]. Furthermore, a meta-analysis of prospective cohort studies also found that obesity was a risk factor that significantly increased diabetic retinopathy [68]. We did not find obesity to be a risk factor since 64.7% of the entire Navajo population was obese, and 72% of the Goshute population was obese. Diabetes, hypertension and retinopathy are multifactorial conditions. Because of this, understanding risk factors for a diagnosis of these diseases can be both challenging and inconsistently reliable, and multiple strategies have been implemented to determine critical factors for disease diagnosis [28,41,69]. Employing a homogenous population to understand these multifactorial conditions may be beneficial and offer more informative results.

Rural populations have greater challenges in accessing quality medical care and preventative care services [69,70,71,72]. Therefore, it is better to understand their unique community risks to provide the most targeted and effective screening strategies to achieve improved health quality within these populations. In the work presented here, we found that diabetic retinopathy was the most prevalent retinal condition in the Navajo. Findings from research studies have supported the notion that geographically isolated populations have a higher prevalence of diabetes type II and have more complications from the disease [7,8,9,10,11,12,13,14,15,16]. Within Australia’s indigenous population, diabetes is three times as great compared to the non-indigenous people of Australia, which was 37.11% [73]. Another geographically isolated population found to have a higher prevalence of diabetes than the overall population is the First Nation population in Alberta, Canada [74]. This population has a 76–87% lifetime risk for the development of chronic disease [74]. Geographic isolation may play a role in the barriers associated with managing the disease. These populations do not have access to resources that could reduce disease complications, including hospitalizations, morbidity and mortality [75,76,77,78]. Within geographically isolated areas of the United States, diabetes rates are higher for geographically isolated residents than the national rates of diabetes [79]. The Southwest Rural Health Research Center determined that the rates of diabetes were 10.6% greater in populations residing in the southeastern region of the United States than the United States national rate of diabetes [80]. Within the Appalachian region of North America, all-cause mortality is 32% higher for the Appalachian region than non-Appalachia, including diabetes [79].

Diabetes type II is a leading cause of death in the American Indian population [80] compared to the general United States population. Almost 16% of the American Indian population was diagnosed with diabetes than non-Hispanic whites, with 8.7% prevalence. The American Indian population has a greater rate of age-adjusted diabetes of all racial and ethnic minority groups [81,82]. Our study population found that the American Indian study participants had a diabetes prevalence of 49.5% (62.2% for the Navajo American Indian study participants and 38% for the Goshute American Indian study participants). This is within the range previously reported and supports the notion that prevalence differs by American Indian tribe [28,29,30,31]. The prevalence of diabetes in both locations is more significant than the geographically isolated populations previously discussed above in other parts of the United States and the world. Our study population only included adults, and this may be the reason why the average age of those with diabetes was 51.6 (18.90–77.98). All individuals who have diabetes are at risk of developing diabetic retinopathy, which was the most prevalent blinding eye disease found within the Navajo American Indian study population. Within our Navajo American Indian study population, the prevalence of diabetic retinopathy was 13.3%, which is lower than the 29% that has been previously reported within the Navajo of Northeast Arizona that was insulin-dependent [56]. Though the prevalence is lower in Arizona, DR still causes microvascular retinal damage and is indicative that the patient needs to manage their diabetes better. Though our prevalence of diabetic retinopathy may be lower than previously published research of 29%, in 60 Navajo American Indians of Northeastern Arizona, it is essential to note that this study was published over thirty years ago [57]. Furthermore, when the previously published study was conducted, the fundus imaging technology was not as advanced as current fundus imaging technology. We believe that this study moves the field forward. Much of the literature focusing on blinding eye disease within the Navajo American Indian population is 20 years or older [56,57] and has yet to be published within the Goshute American Indians at the individual tribal level [50].

Hypertension is also a current challenge for global public health [83,84,85]. Around 1 in 3 adults living in the United States have hypertension [86]. Like diabetes, many people who have hypertension do not know that they have this chronic disease [86]. Moreover, within the United States, only around 37% of those with the disease have the disease controlled using medications and modifying lifestyle factors [85]. For populations that are underserved and geographically isolated, it can be challenging to manage the disease because of limitations to access to care and the ability to make lifestyle modifications. A survey that only included Appalachia residents determined that 47% had hypertension [87]. Within the rural population of the Jilin province in China, rural residents had a 25.93% prevalence of hypertension, which was significantly higher than the urban residents of the same province at 22.73% [88]. Similarly, in the rural areas of Xinjiang, China, the residents have a higher prevalence of hypertension as compared to the national prevalence of hypertension in China. Residents of the rural areas have a prevalence of 32.1% compared to 25.2% [89].

American Indian adults in the United States are 20% more likely to have hypertension than non-Hispanic white adults [23,90]. Hypertension is less studied than diabetes within the American Indian population in the United States [91]. We found that, within this Native American study participants, 41.1% had hypertension (51.1% in the Navajo American Indian study population and 32.0% in the Goshute American Indian population), which was higher than the prevalence of diabetes. Similarly, outcomes due to hypertension can also affect the eye and oral health [92,93]. All individuals who have hypertension are at risk of developing hypertensive retinopathy. Hypertensive retinopathy can cause blindness if not appropriately monitored by an eye care professional [94,95,96,97,98].

Our study found that diabetic retinopathy was the primary eye disease affecting the Four Corners of the Intermountain West’s study population. The finding of diabetic retinopathy in the Four Corners study population is supported by our observation that all study participants with diabetic retinopathy have a medical diagnosis of diabetes, are age 45 years and older and have a hypertension diagnosis. These are all clinical predictors of a diagnosis of diabetic retinopathy [99,100,101]. After fundus evaluation, we did not find the retinal eye disease hypertensive retinopathy (HTR) in our Four Corners of the Intermountain West population. However, in our initial study, in the Goshute American Indian population, hypertensive retinopathy was the most prevalent eye disease, at 42.0% [51]. This could be because increased triglycerides were associated with hypertensive retinopathy within the Goshute population [51]. In contrast, in the Navajo population, dyslipidemia was not associated with a retinopathy outcome. The association of age with hypertension was unique to the Four Corners and Navajo study population.

In the analysis that solely included American Indian study participants, Goshute American Indians had a greater risk of developing hypertensive or diabetic retinopathy than the Navajo American Indians. This study observed two geographically isolated populations, which is essential to uncover associated disease susceptibility variants for multifactorial diseases [102,103,104,105]. Though our Navajo population only accounted for 0.032% of the total tribal members residing in the Navajo Nation of the Four Corners, our Goshute American Indian study population accounted for 12.22% of the Confederate tribes of the Goshute reservation tribal members. Considering our association with hypertension and retinopathy, we still had 73.84% power to detect an association between diabetes and retinopathy at a 73.67% power within our study population [106,107,108]. Moreover, previous studies, including of Navajo Native Americans, had smaller sample sizes, ranging from 32 to 419 study participants [109,110,111,112]. Our laboratory has successfully used small sample sizes in the past to study blinding eye disease in other isolated populations [51,113,114]. Mistrust amongst the American Indian population of the United States is due to historical events that have caused a lack of participation within clinical and scientific research studies [115,116,117]. Our study also collected blood samples, as noted above, which may have deterred patients from participating in our study given the historical mistrust of genetic data and their uses within the Native American population. The previous study on diabetic retinopathy in the Navajo population consisted of only 60 individuals with no blood collection [55]. A previous study that analyzed blood samples in 59 pregnant diabetic Navajo women within the Indian Health Service also had a small sample size, as noted [109]. Rural, isolated populations, even with a small study sample size, are essential populations to include in research because they are essential in the process of understanding diseases and their components in a homogenous setting that can help to foster advancements in public health [103,104]. Small populations can have decreased differences in terms of environmental variability than other populations that are larger and can be more geographically diverse [112]. Health effects can occur at the local level due to resource availability. These resources can include proximity to nutritious food sources and certain medical advancements, causing health inequity within these rural populations [104]. Subsequently, this can affect both the population’s lifestyle and the environment because these health effects can cause disease [104,105].

We employed community involvement in our research study. We built a community relationship with the Goshute and Navajo American Indian population to conduct this research over several years, using community-based participatory research approaches. Community involvement within research is an essential aspect of conducting meaningful research within underserved populations [118]. If these populations go understudied, they will benefit less from precision medicine and experience health disparities. This is currently being seen in the COVID 19 climate, as minority populations are disproportionally burdened by the disease, particularly within the Navajo population [119].

## 5. Conclusions

Our study supports the importance of understanding differences at the tribal level to help inform service delivery for individual tribes/reservations, even if both reside in the same geographical location—in this case, the Intermountain West. Policymakers and other individuals who are delivering and planning relevant health services will be better informed to best meet individual tribes’ needs. This should allow for maximum utilization and access to healthcare. Healthcare services and prevention methods developed at a broad level may not create the change needed to address health disparities between tribes, so they must be designed and tailored for each tribe.

## Figures and Tables

**Table 1:** Demographics of the total study population of the Four Corners of the Intermountain West and Navajo American Indian study population.

Characteristics	Total Study Population (*n* = 51)	American Indian Study Population (*n* = 45)
Age (%)		
45+	31 (60.8%)	27 (60.0%)
18–44	20 (39.2%)	18 (40.0%)
Average Age	46.68 (18.9–77.9)	46.1 (21.6–77.9)
Sex (%)		
Female	30 (58.8%)	28 (62.2%)
Male	21 (41.2%)	17 (37.8%)
Smoker (%)	6 (11.8%)	6 (13.3%)
Multiple Insurance (%)	19 (37.3%)	19 (42.2%)
Single Insurance (%)	32 (62.8%)	26 (57.8%)
Dyslipidemia (%)	17 (33.3%)	16 (35.6%)
Hypertension	26 (51.0%)	23 (51.1%)
25 ≤ BMI < 30	12 (23.5%)	11 (24.4%)
BMI ≥ 30	33 (64.7%)	31 (68.9%)
Diabetic	31 (60.8%)	28 (62.2%)
Non-Diabetic	20 (39.2%)	17 (37.8%)

**Table 2:** Demographics of diabetics vs. non-diabetics within the total study population of the Four Corners of the Intermountain West.

Characteristics (%)	Diabetic (*n* = 31)	Non-Diabetic (*n* = 20)
Sex (%)		
Female	15 (48.4%)	15 (75.0%)
Male	16 (51.6%)	5 (25.0%)
Average Age	51.6 (18.90–77.98)	39.0 (21.68–68.32)
Age Category (%)		
≥45	24 (77.4%)	7 (35.0%)
<45	7 (22.6%)	13 (65.0%)
Race/Ethnicity (%)		
American Indian	28 (90.3%)	17 (85.0%)
Non-American Indian	3 (9.7%)	3 (15.0%)
Multiple Insurance	11 (35.5%)	8 (40.0%)
Smoker (%)	5 (16.1%)	1 (5.0%)
Hypertension	22 (71.0%)	4 (20.0%)
Dyslipidemia	12 (38.7%)	5 (25.0%)

**Table 3:** Demographics of the hypertension vs. the non-hypertensive population of the total study population of the Four Corners of the Intermountain West.

Demographic	Hypertensive Population (*n* = 26)	Non-Hypertensive Population (*n* = 25)
Age (%)		
Age ≥ 45 years old	23 (88.5%)	17 (68.0%)
Age < 45 years old	3 (11.5%)	8 (32.0%)
Average Age	54.94 (30.70–77.98)	38.09 (18.90–73.20)
Sex (%)		
Female (%)	14 (53.9%)	16 (64.0%)
Male (%)	12 (46.2%)	9 (36.0%)
Race/Ethnicity (%)		
American Indian	23 (88.5%)	22 (88.0%)
Non-American Indian	3 (11.5%)	3 (12.0%)
Smoker (%)	3 (11.54%)	3 (12.0%)
Insurance Type (%)		
Multiple Insurance	10 (38.5%)	9 (36.0%)
Single Insurance	16 (61.5%)	16 (64.0%)
Diabetes (%)	22 (84.6%)	9 (36.0%)
Dyslipidemia (%)	12 (46.2%)	5 (20.0%)

**Table 4:** Univariate and multivariate logistic regression models for diabetes in the total study population of the Four Corners of the Intermountain West.

Risk Factor	Odds Ratio with 95% CI *	Adjusted Odds Ratio with 95% CI **
Hypertension	9.8 (2.6–37.4)	6.2 (1.3–30.8)
Age ≥ 45	3.6 (1.8–22.1)	2.5 (0.5–12.1)
Female	3.2 (0.9–11.0)	3.3 (0.8–14.0)
Smoker	3.5 (0.4–32.2)	***
25 ≤ BMI < 30	0.560 (0.2–2.1)	***
BMI ≥ 30	2.0 (0.6–6.5)	***
Dyslipidemia	1.9 (0.5–6.6)	***
American Indian Descent	1.6 (0.3–9.1)	***
Multiple Insurance	1.2 (0.4–3.9)	***

* Univariate analysis of risk factors that may be associated with diabetes; ** Multivariate analysis of factors that were significant in the univariate analysis; *** Not included in the multivariate analysis, did not meet inclusion requirements. CI, confidence interval

**Table 5:** Univariate and multivariate analysis of diabetic factors in the Navajo American Indian study population of the Four Corners of the Intermountain West.

Risk Factor	Odds Ratio with 95% CI *	Adjusted Odds Ratio with 95% CI **
Hypertension	22.5 (4.1–123.8)	13.6 (2.2–84.3)
Female	2.8 (0.7–10.8)	***
Age ≥ 45	8.8 (2.2–35.0)	3.1 (0.6–16.0)
Smoker	3.478 (0.4–32.7)	***
25 ≤ BMI < 30	0.7 (0.2–2.6)	***
BMI ≥ 30	2.1 (0.6–7.6)	***
Dyslipidemia	2.438 (0.6–9.4)	***
Multiple Insurance	1.374 (0.4–4.6)	***

* Univariate analysis of risk factors that may be associated with diabetes; ** Multivariate analysis of factors that were significant in the univariate analysis; *** Not included in the multivariate analysis, did not meet inclusion requirements. CI, confidence interval

**Table 6:** Univariate and multivariate logistic regression models for hypertension in the total study population of the Four Corners of the Intermountain West.

Risk Factor	Odds Ratio with 95% CI *	Adjusted Odds Ratio with 95% CI **
Diabetes	9.8 (2.6–37.4)	5.9 (1.2–28.1)
Age ≥ 45	16.3 (3.8–70.7)	11.5 (2.3–58.5)
Dyslipidemia	3.4 (1.0–11.9)	3.6 (0.7–19.1)
Smoker	1.0 (0.2–5.5)	***
Female	1.5 (0.5–4.7)	***
Multiple Insurance	0.9 (0.3–2.8)	***
25 ≤ BMI < 30	1.0 (0.3–3.5)	***
BMI ≥ 30	1.5 (0.5–4.8)	***
American Indian Descent	1.0 (0.2–5.7)	***

* Univariate analysis of risk factors that may be associated with hypertension; ** Multivariate analysis of factors that were significant in the univariate analysis; *** Not included in the multivariate analysis, did not meet inclusion requirements. CI, confidence interval

**Table 7:** Univariate and multivariate analysis of hypertension factors in the Navajo American Indian study population of the Four Corners of the Intermountain West.

Risk Factor	Odds Ratio with 95% CI *	Adjusted Odds Ratio with 95% CI **
Age ≥ 45 years old	14.3 (3.2–64.6)	8.1 (1.4–46.8)
Diabetes	22.5 (4.1–123.8)	12.4 (1.9–79.0)
Female	2.4 (0.7–8.5)	***
Multiple Insurance	0.9 (0.3–2.9)	***
Dyslipidemia	3.1 (0.9–11.3)	2.8 (0.5–16.6)
Smoker	1.0 (0.2–5.3)	***
25 ≤ BMI < 30	1.2 (0.3–4.7)	***
BMI ≥ 30	1.1 (0.3–3.8)	***

* Univariate analysis of risk factors that may be associated with hypertension; ** Multivariate analysis of factors that were significant in the univariate analysis; *** Not included in the multivariate analysis, did not meet inclusion requirements. CI, confidence interval

**Table 8:** Eye disease prevalence within the total study population.

Eye Disease Diagnosis	Total Study Population (*n* = 51)
Diabetic Retinopathy	6 (11.8%)
Glaucoma	2 (3.9%)
Cataracts	2 (3.9%)

**Table 9:** Univariate and multivariate logistic regression models for diabetic retinopathy in the Navajo American Indian study population.

Risk Factor	Odds Ratio and 95% CI *	Adjusted Odds Ratio with 95% CI **
Age ≥ 45 years old	-	-
Diabetes	-	-
Hypertension	-	-
BMI ≥ 30	0.2 (0.0–1.1)	0.2 (0.0–1.4)
25 ≤ BMI < 30	3.9 (0.7–23.0)	***
Female	1.8 (0.3–10.1)	***
Dyslipidemia	0.9 (0.1–5.5)	***
Multiple Insurances	0.7 (0.1–3.9)	***
Smoke	1.4 (0.1–14.2)	***

All participants with this risk factor had the outcome. * Univariate analysis of risk factors that may be associated with hypertension; ** Multivariate analysis of factors that were significant in the univariate analysis; *** Not included in the multivariate analysis, did not meet inclusion requirements. CI, confidence interval

**Table 10:** Demographic characteristics of the total study population in Ibupah, Utah.

Characteristics	Total Study Population (*n* = 64)	American Indian Study Population (*n* = 50)
Age (%)		
45+	36 (56.3%)	26 (52.0%)
18–44	28 (43.8%)	24 (48.0%)
Average Age	46.24 (22.77–94.21)	44.3 (22.77–78.68)
Sex (%)		
Female	26 (40.6%)	19 (38.0%)
Male	38 (59.4%)	31 (62.0%)
Smoker (%)	36 (56.3%)	32 (64.0%)
Hypertension	19 (29.7%)	16 (32.0%)
25 ≤ BMI < 30	13 (20.3%)	9 (18.0%)
BMI ≥ 30	40 (62.5%)	36 (72.0%)
Diabetic	20 (31.3%)	19 (38.0%)

**Table 11:** Univariate and multivariate analysis of diabetic factors in the Ibupah, Utah study population.

Risk Factors	Odds Ratio and 95% CI *	Adjusted Odds Ratio with 95% CI **
Hypertension	5.0 (1.6–16.3)	5.3 (1.6–16.3)
American Indian Descent	7.4 (0.9–61.2)	7.5 (0.8–68.4)
Multiple Insurance	1.2 (0.3–4.7)	***
Smoker	1.0 (0.3–2.8)	***
Age ≥ 45 years and older	2.2 (0.7–6.9)	***
Female	0.7 (0.2–2.1)	***
25 ≤ BMI < 30	0.5 (0.1–2.1)	***
BMI ≥ 30	3.2 (0.8–13.1)	***

* Univariate analysis of risk factors that may be associated with diabetes; ** Multivariate analysis of factors that were significant in the univariate analysis; *** Not included in the multivariate analysis, did not meet inclusion requirements. CI, confidence interval

**Table 12:** Univariate analysis of diabetic factors in the Goshute American Indian study population of Ibupah, Utah of the Intermountain West.

Risk Factor	Odds Ratio with 95% CI *	Adjusted Odds Ratio with 95% CI **
Hypertension	6.9 (1.8–25.8)	6.1(1.6–23.4)
Age ≥ 45 years and older	3.0 (0.9–10.0)	2.2 (0.6–8.2)
Female	0.5 (0.2–1.7)	***
Multiple Insurances	1.2 (0.3–5.3)	***
Smoker	0.7 (0.2–2.1)	***
25 ≤ BMI < 30	0.7 (0.1–3.0)	***
BMI ≥ 30	1.9 (0.4–8.4)	***

* Univariate analysis of risk factors that may be associated with diabetes; ** Multivariate analysis of factors that were significant in the univariate analysis; *** Not included in the multivariate analysis, did not meet inclusion requirements. CI, confidence interval

**Table 13:** Univariate analysis of hypertension factors in the Ibupah, Utah study population of the Intermountain West.

Risk Factors	Odds Ratio with 95% CI *	Adjusted Odds Ratio with 95% CI **
Diabetes	5.0 (1.6–16.3)	3.9 (1.1–13.6)
Age ≥ 45 years of age	2.8 (0.9–9.2)	2.4 (0.6–8.9)
Female	0.5 (0.1–1.4)	***
Multiple Insurances	1.1 (0.3–4.5)	***
American Indian Descent	1.5 (0.3–6.1)	***
Smoker	1.136 (0.4–3.4)	***
25 ≤ BMI < 30	0.267 (0.1–1.4)	***
BMI ≥ 30	3.4 (0.8–13.8)	2.7(0.6–11.8)

* Univariate analysis of risk factors that may be associated with hypertension; ** Multivariate analysis of factors that were significant in the univariate analysis; *** Not included in the multivariate analysis, did not meet inclusion requirements. CI, confidence interval

**Table 14:** Univariate and multivariate analysis of hypertension factors in the Goshute American Indian study population of Ibupah, Utah.

Risk Factors	Odds Ratio with 95% CI *	Adjusted Odds Ratio with 95% CI **
BMI ≥ 30	-	-
25 ≤ BMI < 30	^	^
Diabetes	6.9 (1.8–25.8)	5.6 (1.3–24.6)
Smoker	1.0 (0.3–3.3)	***
Female	0.4 (0.1–1.5)	***
Multiple Insurances	0.5 (0.1–2.6)	***
Age ≥ 45 years of age	2.6 (0.7–9.3)	***

All Goshute American Indian study participants in Ibupah, Utah with a diagnosis of hypertension had this factor. All Goshute American Indian study participants in Ibupah, Utah did not have this factor. – all in this range; ^ No participants in this range. * Univariate analysis of risk factors that may be associated with hypertension; ** Multivariate analysis of factors that were significant in the univariate analysis; *** Not included in the multivariate analysis, did not meet inclusion requirements. CI, confidence interval

**Table 15:** Analysis of diabetic factors in the overall combined study population.

Risk Factor	Adjusted Odds Ratio with 95% CI ****
HBP	6.1 (2.2–16.8)
Location	3.0 (1.1–7.9)
Sex	1.6 (0.6–4.2)
Age ≥ 45	2.2 (0.8–6.0)
BMI ≥ 25	1.0 (0.1–6.1)
American Indian	7.2 (1.3–39.4)

**** Multivariate analysis of factors HBP, location, sex, age ≥ 45, BMI ≥ 25 and American Indian descent. CI, confidence interval. HBP, high blood pressure

**Table 16:** Analysis of diabetic factors in the overall combined American Indian study population.

Risk Factor	Adjusted Odds Ratio with 95% CI ****
HBP	7.9 (2.7–23.0)
Location	2.2 (0.7–6.3)
Sex	0.9 (0.3–2.8)
Age ≥ 45	2.6 (0.9–7.5)
BMI ≥ 25	3.4 (0.2–69.5)

**** Multivariate analysis of factors HBP, location, sex, age ≥ 45 and BMI ≥ 25 CI, confidence interval. HBP, high blood pressure.

**Table 17:** Analysis of hypertensive factors in the overall combined study population.

Risk Factor	Adjusted Odds Ratio with 95% CI ****
Diabetes	6.1 (2.2–16.9)
Location	1.3 (0.5–3.6)
Sex	0.6 (0.2–1.5)
Age ≥ 45	5.1 (1.8–14.3)
BMI ≥ 25	2.0 (0.3–13.7)
American Indian	0.6 (0.1–2.9)

**** Multivariate analysis of factors HBP, location, sex, age ≥ 45, BMI ≥ 25 and American Indian descent. CI, confidence interval.

**Table 18:** Analysis of hypertensive factors in the overall combined American Indian study population.

Risk Factor	Adjusted Odds Ratio with 95% CI ****
Diabetes	7.8 (2.7–22.8)
Location	1.3 (0.4–3.8)
Sex	0.8 (0.3–2.2)
Age ≥ 45	3.8 (1.3–11.4)
BMI ≥ 25	1.2 (0.1–30.0)

**** Multivariate analysis of factors HBP, location, sex, age ≥ 45, BMI ≥ 25, CI, confidence interval

**Table 19:** Analysis of retinopathy factors in the combined overall study population.

Risk Factor	Adjusted Odds Ratio with 95% CI ****
HBP	3.7 (1.1–12.4)
Diabetes	2.1 (0.6–7.5)
Location	0.1 (0.0–0.2)
Sex	0.5 (0.2–1.6)
Age ≥ 45	1.9 (0.6–6.0)
BMI ≥ 25	1.5 (0.2–11.5)
American Indian	1.2 (0.3–6.1)

**** Multivariate analysis of factors HBP, location, sex, age ≥ 45, BMI ≥ 25 and American Indian descent. CI, confidence interval.

**Table 20:** Analysis of retinopathy factors in the overall combined American Indian study population.

Risk Factor	Adjusted Odds Ratio with 95% CI ****
HBP	2.9 (0.8–10.3)
Diabetes	2.6 (0.7–9.2)
Location	0.1 (0.0–0.2)
Sex	0.8 (0.3–2.4)
Age ≥ 45	1.5 (0.4–4.8)
BMI ≥ 25	0.5 (0.0–7.4)

**** Multivariate analysis of factors HBP, location, sex, age ≥ 45, BMI ≥ 25. CI, confidence interval.
